# Relationship between first trimester physical activity and premature rupture of membranes: a birth cohort study in Chinese women

**DOI:** 10.1186/s12889-024-18791-5

**Published:** 2024-06-28

**Authors:** Chuanzhu Lv, Qian Lu, Caihong Zhang, Shijiao Yan, Huanjun Chen, Xiong-Fei Pan, Chao Fu, Rixing Wang, Xingyue Song

**Affiliations:** 1 Emergency Medicine Center, Sichuan Provincial People’s Hospital, University of Electronic Science and Technology of China, Chengdu, 610072 Sichuan China; 2 Research Unit of Island Emergency Medicine, Hainan Medical University, Chinese Academy of Medical Sciences, (No. 2019RU013), Haikou, 570100 Hainan China; 3 School of Public Health, Hainan Medical University, Haikou, 570100 Hainan China; 4 International school of Nursing, Hainan Medical University, Haikou, 570100 China; 5 Section of Epidemiology and Population Health, Department of Gynecology and Obstetrics, Ministry of Education Key Laboratory of Birth Defects and Related Diseases of Women and Children & National Medical Products Administration Key Laboratory for Technical Research on Drug Products In Vitro and In Vivo Correlation, West China Second University Hospital, Sichuan University, Chengdu, 610041 Sichuan China; 6 West China Biomedical Big Data Center, West China Hospital, Sichuan University, Chengdu, 610041 Sichuan China; 7 Shuangliu Institute of Women’s and Children’s Health, Shuangliu Maternal and Child Health Hospital, Chengdu, 610200 Sichuan China; 8 Center for Epidemiology and Population Health, Integrated Traditional Chinese and Western Medicine Institute & Chengdu Integrated Traditional Chinese and Western Medicine Hospital, Chengdu University of Traditional Chinese Medicine, Chengdu, 610041 Sichuan China; 9 Department of Emergency, Hainan Clinical Research Center for Acute and Critical Diseases, The Second Affiliated Hospital of Hainan Medical University, Haikou, 570100 Hainan China

**Keywords:** Physical activity, Premature rupture of membranes, Pregnant women, Pregnancy, Cohort study

## Abstract

**Objective:**

This study aimed to examine prospective associations of different intensity levels and types of physical activity (PA) in early pregnancy with premature rupture of membranes (PROM) among Chinese pregnant women.

**Methods:**

A total of 6284 pregnant women were included from the Tongji-Shuangliu Birth Cohort. Household/caregiving, occupational, sports/exercise and transportation activities during early pregnancy were investigated by the pregnancy physical activity questionnaire (PPAQ), and the diagnosis of PROM was ascertained during the whole pregnancy. Multivariate logistic regression models were used to estimate the odds ratios (ORs) and 95% confidence interval (CI) for the associations between PA and PROM.

**Results:**

Among the 6284 pregnant women, 1246 were identified to have PROM (19.8%). Women undertaking the highest level (3 third tertile) of PA during pregnancy appeared to have a lower risk of PROM [OR = 0.68, 95%CI 0.58–0.80) when compared to those at the lowest tertile of PA. Similarly, women with increased levels of light intensity activity, moderate-vigorous intensive, household/caregiving activity and meeting exercise guidelines during pregnancy were associated with reduced risks of PROM (OR = 0.69, 95% CI 0.59–0.81, OR = 0.70, 95% CI 0.60–0.82, OR = 0.62, 95% CI 0.53–0.73 and OR = 0.82, 95% CI 0.70–0.97, respectively).

**Conclusions:**

High levels of PA of different intensities and PA of household/caregiving activities and meeting exercise guidelines during the first trimester were associated with a lower incidence of PROM.

**Trial registration:**

The data of human participants in this study were conducted in accordance with the Helsinki Declaration. This study has been approved by the Ethics Committee of Tongji Medical College, Huazhong University of Science and Technology, Wuhan, China ([2017] No. S225). All participants provided written informed consent prior to enrollment. A statement to confirm that all methods were carried out in accordance with relevant guidelines and regulations.

**Supplementary Information:**

The online version contains supplementary material available at 10.1186/s12889-024-18791-5.

## Introduction

Premature rupture of membranes (PROM) refers to the spontaneous rupture of fetal membranes before the onset of labor. It can be divided into preterm premature rupture of membranes (PPROM) and term premature rupture of membranes (TPROM) [1, 2]. The incidence of PROM in all deliveries is about 5-10% worldwide [3, 4], and 12.07% in China [5]. The incidence of TPROM is about 8% [6], while PPROM occurs in 2.0 to 3.5% of pregnant women [4]. PROM is the greatest risk factor for the fetus preterm birth complications respiratory distress, neurodevelopmental disorders, neonatal white matter damage and death [1, 7, 8].

The etiology of PPROM is multifactorial and its pathophysiology and pathogenesis have not been fully elucidated. Studies have shown that PROM is a membrane disorder, and pregnancy increases oxidative stress (OS), leading to systemic inflammation and the production of reactive oxygen species (ROS) [9]. Inflammation and OS are inseparable, which together induce membrane weakening, aging and damage [10–13].

In recent years, guidelines around the world have recommended that pregnant women with no contraindications perform Physical Activity (PA) throughout pregnancy and postpartum, mainly through moderate-intensity aerobic and resistance training activities of no less than 150 min per week, which can also be yoga or stretching or pelvic floor muscle training. Research shows that moderate PA can reduce the risk of pregnancy weight gain, gestational diabetes (GDM), preeclampsia (PE), postpartum depression, and cesarean Sects. [14–19].Meanwhile, PA can be used as a preventive or therapeutic measure, with benefits for the mother, fetus and newborn. Studies have shown that regular moderate and low-intensity aerobic exercise can reduce or eliminate OS [20, 21].

At present, most studies on PROM focus on its clinical characteristics, complications, influencing factors and treatment decisions [22–24]. Previous studies have shown that PA during pregnancy is essential for maternal and infant health [25–27], and that moderate to low intensity PA during pregnancy can reduce the risk of PROM [28]; however, some other studies have failed to find a significant association [29]. At present, there are few studies on the relationship between physical activity during pregnancy and premature rupture of membranes in China. Therefore, we conducted a prospective cohort study to investigate the relationship between PA levels, intensity and PROM in early pregnancy in Chinese pregnant women.

## Materials and methods

### Design and population/study design and participants

Data were obtained from the Tongji-Shuangliu Birth Cohort (TSBC). Detailed study design and procedures have been previously reported [30]. Pregnant women who had their first antenatal visit at the hospital were invited to participate in our research project. Included in our research project if the following criteria are met: (1) Pregnant women aged 18–40 years and carrying a singleton; (2) gestational age ≤ 15 weeks. Women with the following conditions were excluded: (1) Pregnant women have received assisted reproductive technology (such as in vitro fertilization or intrauterine fertilization); (2) Pregnant women have serious chronic diseases or infectious diseases (such as cancer, AIDS or tuberculosis); (3) Pregnant women are unable to fill out the questionnaire or refuse to sign Informed consent [30].

Baseline recruitment began in 2017 and ended in 2020, and a total of 7,281 pregnant women were enrolled in our study during the early stages of pregnancy. we excluded women with the following conditions: 2 women had inconsistent age information, 8 women with missing data on educational level, and 987 women were given birth outside the hospital. In the end, 6284 women with complete data were included in the analyses. This study was approved by the Ethics Committee of Tongji Medical College, Huazhong University of Science and Technology (Wuhan, China). ALL participants provided written informed consent at the time of first recruitment.

### Physical activity assessments

PA was assessed using the Chinese version of the Pregnancy Physical Activity Questionnaire (PPAQ) at baseline. The Chinese version of the PPAQ showed good reliability and validity in Chinese pregnant women [31]. The PPAQ contained a total of 32 activities, including household/caregiving activities (13 activities), occupational activities (5 activities), sports/exercise (8 activities), transportation (3 activities), and inactivity (3 activities) [31, 32]. These activities can be divided into sedentary activities (≤ 1.5MET), light activities (1.5-2.9MET), moderate activities (3.0-6.0MET), and vigorous activities (> 6.0MET) [31, 32]. The energy expenditure of sedentary activities is not used to calculate total PA. In addition, the average weekly total energy expenditure (MET-h/week) can be calculated by multiplying the time spent on each activity by the corresponding intensity of each activity. Determine the activity intensity according to the Program of Physical Activity [33].

The PA estimates of different intensities and different categories were divided into tertiles, and the first tertile was used as the reference group. As few pregnant women engaged in vigorous intensity PA, moderate intensity PA and vigorous intensity PA were combined into the moderate-vigorous physical activity (MVPA) group.

WHO guidelines on physical activity recommend that pregnant women with no contraindications to exercise do at least 150 min of moderate-intensity aerobic exercise per week. Therefore, if participants spent more than 7.5 h per week on moderate to vigorous intensity sports/exercise, the guidelines were met, so we classified PA as " yes " or " no “ [19].

### PROM assessments

PROM is rupture of membranes before the onset of labor. Membrane rupture before labor and before 37 weeks of gestation is referred to as preterm PROM. PROM was diagnosed based on the pregnant woman’s medical history and physical examination. The diagnosis of premature rupture of membranes is usually through the flow of amniotic fluid from the cervical canal and into the vagina, and then through the detection of the pH value of the vaginal fluid to diagnose [34]. The normal pH of vaginal secretions is generally 4.5-6.0, while amniotic fluid is usually pH 7.1–7.3 [34].

### Assessments of covariates

Participants were invited to participate in baseline interviews to complete structured questionnaires about their lifestyle information, including sociodemographic information, PA, smoking, marital and reproductive history, and past medical history. Interviews were conducted by trained professional investigators through face-to-face interviews to minimize potential language and literacy barriers. Pre-pregnancy weight was self-reported by pregnant women, and pre-pregnancy BMI (kg/m2) was calculated by dividing weight (kg) by height (m) squared. Pre-pregnancy BMI was divided into 4 categories according to the Chinese standards: underweight (< 18.5 kg/m2), normal weight (18.5–23.9 kg/m2), overweight (24.0–27.9 kg/m2), and obese (≥ 28.0 kg/m2) [35].

### Statistical analyses

Baseline characteristics were presented as mean (standard deviation) for continuous variables, and N (%) for categorical variables. The chi-square test was used to compare the differences of general characteristics between PROM and non-PROM pregnant women. Chi-square test and Wilcoxon rank sum test were used to compare the energy expenditure of different intensity and different types of PA between PROM and Non-PROM pregnant women. Multivariable logistic regression analysis was used to determine the association between PA and PROM. By referring to the relevant literature, we adjusted for potential confounders in sequential steps [36–44]. Model 1 is unadjusted. Model 2 adjusted for maternal age (continuous), occupations (farmers, workers, service worker, office and technical staff, housewife or unemployed), education level (senior high school or below, above senior high school), smoking status (never, ever, current), yearly income (<50,000, ≥ 50,000), pre-pregnancy BMI (continuous), GDM during pregnancy, HDP during pregnancy, vaginitis before pregnancy. Model 3 additionally adjusted for infant sex (male, female) and gestational weeks (continuous).

Stratified analyses were performed according to GDM during pregnancy, HDP during pregnancy and vaginitis before pregnancy. Tests for interaction were conducted by adding interaction terms of the research variable and the stratifying variable in the final model. SPSS 25.0 software was used for all analyses, with two-sided *P* values of 0.05 as the significance level.

## Results

### Baseline characteristics of study participants

Among 6284 pregnant women with complete data included in the analyses, 1246 (19.8) incident case of PROM were identified. Mean age was 26.6 years (standard deviation, 3.7 years), mean pre-pregnancy BMI was 21.0 kg/m2 (standard deviation, 3.0 kg/m2), and mean gestational age was 38.9 weeks (standard deviation,1.2 weeks). Compared with their counterparts, women who developed PROM were more likely to be younger, have a shorter gestational weeks BMI and more likely to have vaginitis during pregnancy (Table 1).

**Table 1:** Characteristics of study participants by premature rupture of membranes

Characteristic	Total	PROM	Non-PROM	*P* value
Maternal age, years	26.6 (3.7)	26.3 (3.5)	26.6 (3.7)	0.001
Gestational weeks	38.9 (1.2)	38.5 (1.3)	39.0(1.1)	0.000
Infant sex, n (%)				0.226
Male	3222 (51.3)	658 (52.8)	2564 (50.9)	
Female	3062 (48.7)	588 (47.2)	2474 (49.1)	
Occupations				0.388
Farmers	24 (0.4)	3 (0.2)	21 (0.4)	
Workers	88 (1.4)	16 (1.3)	72 (1.4)	
Service worker	745 (11.9)	139 (11.2)	606 (12.0)	
Office and technical staff	2095 (33.3)	442 (35.5)	1653 (32.8)	
Housewife/unemployed	3332 (53.0)	646 (51.8)	2686 (53.3)	
Education level, n (%)				0.071
Senior high school or below	3662 (58.3)	698 (56.0)	2964 (58.8)	
Above senior high school	2622 (41.7)	548 (44.0)	2074 (41.2)	
Smoking status, n (%)				0.276
Current	109 (1.7)	18 (1.4)	91 (1.8)	
Former	306 (4.9)	70 (5.6)	236 (4.7)	
Never	5869 (93.4)	1158 (92.9)	4711 (93.5)	
Yearly income, yuan				0.072
<50,000	2719 (43.3)	511 (41.0)	2208 (43.8)	
≥ 50,000	3565 (56.7)	735 (59.0)	2830 (56.2)	
Pre-pregnancy BMI, kg/m^2^	21.0 (3.0)	20.8 (2.9)	21.0 (3.0)	0.646
GDM				0.279
No	5825 (92.7)	1164 (93.4)	4660 (92.5)	
Yes	459 (7.3)	82 (6.6)	378 (7.5)	
HDP				0.545
No	6142 (97.7)	1215 (97.5)	4927 (97.8)	
Yes	142 (2.3)	31 (2.5)	111 (2.2)	
Vaginitis				0.023
No	5332 (84.9)	1083 (86.9)	4249 (84.3)	
Yes	952 (15.1)	163 (13.1)	789 (15.7)	

*Note* Data are shown as mean (SD) for continuous variables, and N (%) for categorical variables

*Abbreviations* PROM, premature rupture of membranes; BMI, body mass index; GDM, gestational diabetes mellitus; HDP, hypertensive disorders of pregnancy

### Energy expenditure of PA in pregnant women with PROM and PPROM

The physical activity levels of the study participants are presented in Table 2. The median total weekly energy expenditure of 6284 pregnant women was 119.5 MET-h/week. Type of activity is mainly sedentary and light PA, and pregnant women expended the most energy during household/caregiving activities. There was no difference between the two groups in the level of total PA and all types of PA.

**Table 2:** Physical activity during pregnancy by PROM

Physical activity (MET-h/week)	Total (*n* = 6284)	Non-PROM (*n* = 5038)	PROM (*n* = 1246)
Total physical activity	119.5 (72.6-173.4)	118.9 (72.5-173.7)	121.2 (74.0-172.0)
By intensity			
Sedentary	34.0 (17.5–79.6)	34.1 (17.5–78.8)	33.8 (17.5–80.9)
Light	38.7 (21.0-66.3)	38.7 (20.7–66.3)	39.7 (21.5–66.3)
Moderate-Vigorous	23.7 (10.1–46.7)	23.5 (10.1–46.7)	25.2 (11.0-46.8)
By domain			
Household/caregiving	27.4 (12.1–56.4)	27.8 (11.9–56.4)	26.1 (12.1–56.6)
Occupational	0 (0-71.1)	0 (0-70.1)	0 (0-71.1)
Sports/exercise	4.2 (0.8–9.6)	3.8 (0.8–9.60)	4.4 (1.5–9.6)
Transportation	14.0 (7.0-26.3)	14.0 (7.0-26.3)	15.8 (7.0–28.0)

*Note* Data are shown as median (IQR) for continuous variables

*Abbreviations* IQR, interquartile range; PROM, premature rupture of membranes; MET, metabolic equivalent. Differences between groups were assessed using Mann-Whitney U test

### Relationship between PA and PROM

The associations between PA in early pregnancy and PROM are presented in Table 3. Compared with the first tertile of total PA, the risk of PROM in the third tertile was reduced by 32% (OR:0.68,95%CI: 0.58–0.80) after full adjustment for covariates. In particular, the highest tertile of light PA, moderate-vigorous PA and household/caregiving PA was associated with a 31% (OR: 0.69, 95% CI: 0.59–0.81), 30% (OR: 0.70, 95% CI: 0.60–0.82) and 38% (OR: 0.62, 95% CI: 0.53–0.73) lower risk of GDM respectively, compared with the lowest tertile. Similar findings were obtained in the stratified analyses, while the magnitude of the association between PA level and PROM was significantly stronger in one stratum than the other (Appendix Tables 1, 2 and 3).

**Table 3:** Odds ratios for PROM associated with the levels of PA

Physical activity (MET-h/week)	Case(*n*)	Model 1^a^	Model 2^b^	Model 3^c^
		OR (95% CI)	OR (95% CI)	OR (95% CI)
Total physical activity				
1st tertile	469	1.00	1.00	1.00
2nd tertile	440	0.99 (0.86–1.15)	0.94 (0.80–1.09)	0.93 (0.80–1.08)
3rd tertile	337	0.78 (0.67–0.91)	0.69 (0.59–0.80)	0.68 (0.58–0.80)
Sedentary				
1st tertile	389	1.00	1.00	1.00
2nd tertile	419	0.91 (0.78–1.06)	0.89 (0.76–1.04)	0.89 (0.76–1.05)
3rd tertile	438	1.07 (0.92–1.25)	1.02 (0.83–1.26)	1.06 (0.86–1.31)
Light				
1st tertile	346	1.00	1.00	1.00
2nd tertile	429	0.87 (0.75–1.01)	0.89 (0.76–1.03)	0.87 (0.75–1.01)
3rd tertile	471	0.68 (0.58–0.79)	0.70 (0.60–0.82)	0.69 (0.59–0.81)
Moderate-Vigorous				
1st tertile	341	1.00	1.00	1.00
2nd tertile	413	0.93 (0.80–1.08)	0.93 (0.80–1.08)	0.93 (0.80–1.09)
3rd tertile	492	0.68 (0.59–0.80)	0.70 (0.60–0.81)	0.70 (0.60–0.82)
Household/caregiving				
1st tertile	325	1.00	1.00	1.00
2nd tertile	408	0.83 (0.72–0.96)	0.85 (0.74–0.99)	0.84 (0.73–0.98)
3rd tertile	513	0.60 (0.51–0.70)	0.63 (0.54–0.74)	0.62 (0.53–0.73)
Occupational				
1st tertile	678	1.00	1.00	1.00
2nd tertile	181	1.11 (0.93–1.34)	1.20 (0.92–1.57)	1.18 (0.91–1.55)
3rd tertile	387	1.13 (0.98–1.29)	1.21 (0.95–1.54)	1.23 (0.96–1.57)
Sports/exercise				
1st tertile	393	1.00	1.00	1.00
2nd tertile	428	0.92 (0.79–1.06)	0.92 (0.79–1.07)	0.92 (0.79–1.07)
3rd tertile	425	0.87 (0.75–1.02)	0.88 (0.75–1.03)	0.88 (0.76–1.03)
Transportation				
1st tertile	371	1.00	1.00	1.00
2nd tertile	425	0.96 (0.82–1.11)	0.95 (0.82–1.10)	0.94 (0.81–1.10)
3rd tertile	450	0.93 (0.80–1.08)	0.93 (0.80–1.09)	0.96 (0.82–1.13)
Meeting PA guidelines ^d^				
No	390	1.00	1.00	1.00
Yes	856	0.82 (0.70–0.97)	0.82 (0.70–0.97)	0.82 (0.70–0.97)

*Abbreviation* PROM, premature rupture of membranes; OR, odds ratio; CI, confidential interval

^a^ Unadjusted odds ratio

^b^ Adjusted for maternal age (continuous), occupations (farmers, workers, service worker, office and technical staff, housewife or unemployed), education level (senior high school or below, above senior high school), smoking status (never, ever, current), yearly income (<50,000, ≥ 50,000), pre-pregnancy BMI (continuous), GDM during pregnancy, HDP during pregnancy, vaginitis before pregnancy

^c^ Additionally adjusted for infant sex (male, female), gestational weeks (continuous)

^d^ Meeting WHO guidelines of ≥ 7.5 MET-h/week in sports/exercise activities of moderate-intensity or greater

### Relationship between PA and PPROM

The associations between PA in early pregnancy and PPROM are presented in Table 4. Compared with the first tertile of total PA, the risk of PROM in the third tertile was reduced by 63% (OR:0.37,95%CI: 0.18–0.75) after full adjustment for covariates. In particular, the highest tertile of light PA was associated with a 54% (OR: 0.46 95% CI: 0.24–0.90) lower risk of GDM, compared with the lowest tertile.

**Table 4:** Odds ratios for PPROM associated with the levels of PA

Physical activity (MET-h/week)	Case(*n*)	Model 1^a^	Model 2^b^	Model 3^c^
		OR (95% CI)	OR (95% CI)	OR (95% CI)
Total physical activity				
1st tertile	41	1.00	1.00	1.00
2nd tertile	30	0.89(0.55–1.44)	0.72 (0.45–1.17)	0.68 (0.37–1.26)
3rd tertile	17	0.55 (0.32–0.96)	0.40 (0.22–0.71)	0.37 (0.18–0.75)
Sedentary				
1st tertile	27	1.00	1.00	1.00
2nd tertile	31	1.15 (0.68–1.93)	1.02 (0.59–1.75)	0.65 (0.32–1.30)
3rd tertile	30	1.13 (0.67–1.90)	0.78 (0.39–1.56)	0.85 (0.37–1.95)
Light				
1st tertile	39	1.00	1.00	1.00
2nd tertile	27	0.68 (0.41–1.11)	0.68 (0.41–1.11)	0.56 (0.30–1.05)
3rd tertile	22	0.56 (0.33–0.94)	0.55 (0.32–0.94)	0.46 (0.24–0.90)
Moderate-Vigorous				
1st tertile	37	1.00	1.00	1.00
2nd tertile	30	0.91 (0.56–1.48)	0.92 (0.56–1.49)	1.09 (0.58–2.04)
3rd tertile	21	0.60 (0.35–1.02)	0.60 (0.35–1.03)	0.68 (0.34–1.34)
Household/caregiving				
1st tertile	39	1.00	1.00	1.00
2nd tertile	32	0.89 (0.56–1.43)	0.90 (0.56–1.45)	1.12 (0.62–2.04)
3rd tertile	17	0.45 (0.25–0.80)	0.46 (0.26–0.83)	0.50 (0.24–1.04)
Occupational				
1st tertile	47	1.00	1.00	1.00
2nd tertile	17	1.49 (0.85–2.60)	0.99 (0.44–2.23)	0.95 (0.34–2.59)
3rd tertile	24	0.98 (0.60–1.61)	0.63 (0.29–1.38)	0.78 (0.30–2.01)
Sports/exercise				
1st tertile	29	1.00	1.00	1.00
2nd tertile	36	1.15 (0.70–1.88)	1.16 (0.71–1.90)	1.01 (0.54–1.89)
3rd tertile	23	0.77 (0.44–1.33)	0.77 (0.44–1.34)	0.64 (0.32–1.26)
Transportation				
1st tertile	31	1.00	1.00	1.00
2nd tertile	36	1.19 (0.73–1.93)	1.15 (0.71–1.87)	1.19 (0.65–2.20)
3rd tertile	21	0.77 (0.44–1.35)	0.83 (0.42–1.29)	0.88 (0.44–1.78)
Meeting PA guidelines ^d^				
No	20	1.00	1.00	1.00
Yes	68	0.66 (0.40–1.10)	0.66 (0.40–1.10)	0.55 (0.29–1.04)

*Abbreviation* PPROM, preterm premature rupture of membranes; OR, odds ratio; CI, confidential interval

^a^ Unadjusted odds ratio

^b^ Adjusted for maternal age (continuous), occupations (farmers, workers, service worker, office and technical staff, housewife or unemployed), education level (senior high school or below, above senior high school), smoking status (never, ever, current), yearly income (<50,000, ≥ 50,000), pre-pregnancy BMI (continuous), GDM during pregnancy, HDP during pregnancy, vaginitis before pregnancy

^c^ Additionally adjusted for infant sex (male, female), gestational weeks (continuous)

^d^ Meeting WHO guidelines of ≥ 7.5 MET-h/week in sports/exercise activities of moderate-intensity or greater

## Discussion

In our prospective cohort study, the incidence of PROM and PPROM in pregnant women was 19.8% and 1.4% respectively. In this large prospective cohort study, total intensity PA, light intensity PA, and moderate-to-severe intensity PA energy expenditure were negatively associated with the risk of PROM at different levels of PA. However, PPROM was negatively correlated with total PA and light intensity PA. In addition, energy consumption in family activities was negatively correlated with the prevalence of PROM and PPROM.

The incidence of PROM in all deliveries is about 5-10% worldwide [3, 4]. In China, studies have shown that the incidence of premature rupture of membranes is 12.07% [5].Our study concluded that the incidence of PROM was higher than previous conclusions and that PPROM was lower than previous conclusions. This difference may be due to inconsistencies in the diagnosis of PROM or to individual differences in the subjects themselves. Moderate- and low-intensity PA was found to be associated with a reduced risk of PROM in another study conducted in China [28]. This is similar to our findings, where we found that both total PA and light intensity in the first trimester of pregnancy had a certain effect of reducing PROM, and the results further confirmed the benefit of PA. Although they used another questionnaire [the International Physical Activity Questionnaire (IPAQ-SF)] to investigate PA [45], the similar results we both obtained further suggests that the relationship between PA and PROM is independent of the survey tool.

Previous studies have found that vaginitis caused by infection is a risk factor for premature rupture of membranes [46, 47]. One possible reason is that infection leads to the destruction of the glial fibers of the membranes, which reduces the toughness of the membranes and increases the risk of PROM. GDM found to be associated with increased risk of PROM [47]. Due to poor blood sugar control, abdominal pressure is too large, and the lateral pressure of fetal membrane is also increased, which is easy to lead to PROM. HDP is also a predisposing factor for PROM [48].The basic pathological changes of HDP are manifested as systemic arteriolar spasm and vascular lumen stenosis, resulting in placental vascular degeneration, bleeding, infarction, etc., resulting in placenta and fetus ischemia and hypoxia, thus increasing the risk of PROM.

At present, there is a lot of evidence that adequate PA during pregnancy is beneficial to prevent adverse pregnancy outcomes [29, 49, 50]. The possible biological mechanism is that moderate PA in pregnancy can improve maternal antioxidant capacity and reduce inflammatory markers in maternal blood, which in turn reduce mitochondrial superoxide and hydrogen peroxide in placenta, and protect maternal and fetal health [51, 52]. Therefore, we speculate that insufficient PA during pregnancy may lead to the occurrence of GDM and HDP, and then lead to the occurrence of PROM, which may be related to Oxidative stress (OS). PA and exercise training during pregnancy can promote placental development, reduce pro-inflammatory markers in maternal blood, and can reduce components of the oxidative stress system in the placenta [53–55].

As a result, the new WHO guidelines on physical activity and sedentary behavior in 2020 recommend that pregnant women with no contraindications regularly engage in at least 150 min of moderate-intensity aerobic exercise per week throughout pregnancy and after delivery [19]. Our findings further support this suggestion.

The advantages of this study lie in its large sample size and prospective study design. However, there are some limitations to our study. First, PA is assessed using a PPAQ instead of objective measurements. Although the Chinese version of the PPAQ has been validated in pregnant women in China, measurement errors may still exist. Second, we only evaluated PA in the first trimester and did not evaluate PA in the second and third trimesters, which may cause some errors. Third, the diet of pregnant women was not adjusted, which may be an important confounding factor. Forth, this study concluded that total intensity PA, light intensity PA, and moderate to vigorous intensity PA energy expenditure are protective factors for PROM, so it is reasonable to speculate that pregnant women with higher physical fitness are more physically active. At the same time, healthy pregnant women with high physical fitness may have fewer instances of early PROM, which requires further research. Finally, our study was conducted in one region of China, and future studies need to be conducted in different regional groups to confirm the generality of our work.

## Conclusion

Elevated PA levels in early pregnancy were negatively associated with the risk of PROM. Our findings add to the benefits of PA in preventing PROM. Our study may highlight the need for pregnant women to adopt an active and healthy lifestyle to reduce the risk of PROM.

### Electronic supplementary material

Below is the link to the electronic supplementary material.

Supplementary Material 1

## Data Availability

The datasets used and/or analysed during the current study are available from the corresponding author on reasonable request.

## Abbreviations

PROM: Premature Rupture of Membranes
PPROM: Preterm Premature Rupture Of Membranes
PA: Physical Activity
MVPA: Moderate to Vigorous Physical Activity
