# Supplementary material for: Relationship between first trimester physical activity and premature rupture of membranes: a birth cohort study in Chinese women
Source: BMC Public Health. 2024 Jun 28;24:1736. doi: 10.1186/s12889-024-18791-5 (PMC11214697; doi:10.1186/s12889-024-18791-5)
Supplement: Supplementary file 1 — Supplementary Material 1 [file 12889_2024_18791_MOESM1_ESM.docx]

**Appendix Table 1: Odds ratios for PROM associated with the levels of PA stratified by GDM during pregnancy**

| Physical activity  (MET-h/week) | Non-GDM | |  | GDM | | *P* for interaction |
| --- | --- | --- | --- | --- | --- | --- |
|  |  |  |  |  |  |  |
|  | OR | 95%CI |  | OR | 95%CI |  |
| Total physical activity |  |  |  |  |  | 0.355 |
| 1st tertile | 1.00 |  |  | 1.00 |  |  |
| 2nd tertile | 1.16 | 0.92-1.46 |  | 0.93 | 0.37-2.36 |  |
| 3rd tertile | 1.11 | 0.78-1.57 |  | 0.85 | 0.21-3.56 |  |
| Sedentary |  |  |  |  |  | 0.191 |
| 1st tertile | 1.00 |  |  | 1.00 |  |  |
| 2nd tertile | 0.89 | 0.75-1.06 |  | 0.64 | 0.32-1.26 |  |
| 3rd tertile | 0.8 | 0.60-1.06 |  | 1.85 | 0.56-6.10 |  |
| Light |  |  |  |  |  | 0.259 |
| 1st tertile | 1.00 |  |  | 1.00 |  |  |
| 2nd tertile | 1.00 | 0.82-1.22 |  | 0.65 | 0.27-1.53 |  |
| 3rd tertile | 0.84 | 0.63-1.11 |  | 1.51 | 0.45-5.00 |  |
| Moderate-Vigorous |  |  |  |  |  | 0.044 |
| 1st tertile | 1.00 |  |  | 1.00 |  |  |
| 2nd tertile | 0.96 | 0.81-1.13 |  | 0.82 | 0.43-1.54 |  |
| 3rd tertile | 0.76 | 0.60-0.95 |  | 0.89 | 0.34-2.32 |  |
| Household/caregiving |  |  |  |  |  | 0.102 |
| 1st tertile | 1.00 |  |  | 1.00 |  |  |
| 2nd tertile | 0.88 | 0.73-1.06 |  | 0.61 | 0.29-1.29 |  |
| 3rd tertile | 0.75 | 0.58-0.98 |  | 0.25 | 0.08-0.78 |  |
| Occupational |  |  |  |  |  | 0.304 |
| 1st tertile | 1.00 |  |  | 1.00 |  |  |
| 2nd tertile | 1.11 | 0.87-1.41 |  | 0.72 | 0.25-2.04 |  |
| 3rd tertile | 1.23 | 0.95-1.60 |  | 0.55 | 0.17-1.18 |  |
| Sports/exercise |  |  |  |  |  | 0.936 |
| 1st tertile | 1.00 |  |  | 1.00 |  |  |
| 2nd tertile | 0.97 | 0.83-1.14 |  | 1.08 | 0.58-2.01 |  |
| 3rd tertile | 0.99 | 0.84-1.18 |  | 0.79 | 0.40-1.57 |  |
| Transportation |  |  |  |  |  | 0.169 |
| 1st tertile | 1.00 |  |  | 1.00 |  |  |
| 2nd tertile | 1.03 | 0.87-1.21 |  | 1.10 | 0.56-2.14 |  |
| 3rd tertile | 1.19 | 0.98-1.45 |  | 1.28 | 0.55-3.02 |  |

Abbreviation: PROM, premature rupture of membranes; GDM, gestational diabetes mellitus; OR, odds ratio; CI, confidential interval.

Odds ratios (95% CI) were calculated after adjustment of maternal age (continuous), occupations (farmers, workers, service worker, office and technical staff, housewife or unemployed), education level (senior high school or below, above senior high school),smoking status (never, ever, current) ,yearly income (＜50000，≥50000), pre‐pregnancy BMI (continuous), HDP during pregnancy, vaginitis before pregnancy, infant sex (male, female) and gestational weeks (continuous).

**Appendix Table 2: Odds ratios for PROM associated with the levels of PA stratified by HDP during pregnancy**

| Physical activity  (MET-h/week) | Non-HDP | | HDP | | | *P* for interaction |
| --- | --- | --- | --- | --- | --- | --- |
|  | OR | 95%CI |  | OR | 95%CI |  |
| Total physical activity |  |  |  |  |  | 0.255 |
| 1st tertile | 1.00 |  |  | 1.00 |  |  |
| 2nd tertile | 1.18 | 0.94-1.48 |  | 0.27 | 0.51-1.49 |  |
| 3rd tertile | 1.11 | 0.79-1.56 |  | 0.36 | 0.03-4.36 |  |
| Sedentary |  |  |  |  |  | 0.205 |
| 1st tertile | 1.00 |  |  | 1.00 |  |  |
| 2nd tertile | 0.87 | 0.73-1.03 |  | 1.13 | 0.37-3.44 |  |
| 3rd tertile | 0.81 | 0.61-1.08 |  | 1.47 | 0.22-9.65 |  |
| Light |  |  |  |  |  | 0.535 |
| 1st tertile | 1.00 |  |  | 1.00 |  |  |
| 2nd tertile | 0.98 | 0.80-1.19 |  | 1.03 | 0.24-4.37 |  |
| 3rd tertile | 0.87 | 0.66-1.15 |  | 0.61 | 0.08-4.59 |  |
| Moderate-Vigorous |  |  |  |  |  | 0.036 |
| 1st tertile | 1.00 |  |  | 1.00 |  |  |
| 2nd tertile | 0.95 | 0.81-1.12 |  | 0.89 | 0.27-2.92 |  |
| 3rd tertile | 0.75 | 0.60-0.95 |  | 1.67 | 0.33-8.49 |  |
| Household/caregiving |  |  |  |  |  | 0.045 |
| 1st tertile | 1.00 |  |  | 1.00 |  |  |
| 2nd tertile | 0.87 | 0.72-1.04 |  | 0.62 | 0.15-2.55 |  |
| 3rd tertile | 0.72 | 0.56-0.93 |  | 0.53 | 0.10-2.90 |  |
| Occupational |  |  |  |  |  | 0.433 |
| 1st tertile | 1.00 |  |  | 1.00 |  |  |
| 2nd tertile | 1.11 | 0.87-1.40 |  | 1.05 | 0.15-7.62 |  |
| 3rd tertile | 1.19 | 0.92-1.54 |  | 2.58 | 0.37-17.95 |  |
| Sports/exercise |  |  |  |  |  | 0.782 |
| 1st tertile | 1.00 |  |  | 1.00 |  |  |
| 2nd tertile | 0.97 | 0.83-1.13 |  | 1.77 | 0.50-6.31 |  |
| 3rd tertile | 0.94 | 0.80-1.11 |  | 3.76 | 1.08-13.13 |  |
| Transportation |  |  |  |  |  | 0.161 |
| 1st tertile | 1.00 |  |  | 1.00 |  |  |
| 2nd tertile | 1.03 | 0.87-1.21 |  | 1.32 | 0.42-4.19 |  |
| 3rd tertile | 1.19 | 0.98-1.45 |  | 1.30 | 0.30-5.76 |  |

Abbreviation: PROM, premature rupture of membranes; HDP, hypertensive disorders of pregnancy; OR, odds ratio; CI, confidential interval.

Odds ratios (95% CI) were calculated after adjustment of maternal age (continuous), occupations (farmers, workers, service worker, office and technical staff, housewife or unemployed), education level (senior high school or below, above senior high school),smoking status (never, ever, current) ,yearly income (＜50000，≥50000), pre‐pregnancy BMI (continuous), GDM during pregnancy, vaginitis before pregnancy, infant sex (male, female) and gestational weeks (continuous).

**Appendix Table 3: Odds ratios for PROM associated with the levels of PA stratified by vaginitis before pregnancy**

| Physical activity  (MET-h/week) | Non-Vaginitis | | Vaginitis | | | *P* for interaction |
| --- | --- | --- | --- | --- | --- | --- |
|  | OR | 95%CI |  | OR | 95%CI |  |
| Total physical activity |  |  |  |  |  | 0.269 |
| 1st tertile | 1.00 |  |  | 1.00 |  |  |
| 2nd tertile | 1.22 | 0.96-1.55 |  | 0.76 | 0.40-1.41 |  |
| 3rd tertile | 1.26 | 0.88-1.82 |  | 0.47 | 0.18-1.19 |  |
| Sedentary |  |  |  |  |  | 0.149 |
| 1st tertile | 1.00 |  |  | 1.00 |  |  |
| 2nd tertile | 0.85 | 0.71-1.02 |  | 1.05 | 0.67-1.65 |  |
| 3rd tertile | 0.78 | 0.57-1.05 |  | 1.22 | 0.58-2.56 |  |
| Light |  |  |  |  |  | 0.370 |
| 1st tertile | 1.00 |  |  | 1.00 |  |  |
| 2nd tertile | 0.97 | 0.79-1.20 |  | 0.94 | 0.55-1.62 |  |
| 3rd tertile | 0.83 | 0.62-1.12 |  | 1.01 | 0.46-2.20 |  |
| Moderate-Vigorous |  |  |  |  |  | 0.017 |
| 1st tertile | 1.00 |  |  | 1.00 |  |  |
| 2nd tertile | 0.92 | 0.77-1.09 |  | 1.21 | 0.77-1.91 |  |
| 3rd tertile | 0.71 | 0.55-0.90 |  | 1.15 | 0.63-2.11 |  |
| Household/caregiving |  |  |  |  |  | 0.031 |
| 1st tertile | 1.00 |  |  | 1.00 |  |  |
| 2nd tertile | 0.92 | 0.77-1.09 |  | 0.87 | 0.53-1.41 |  |
| 3rd tertile | 0.71 | 0.55-0.90 |  | 0.92 | 0.46-1.82 |  |
| Occupational |  |  |  |  |  | 0.507 |
| 1st tertile | 1.00 |  |  | 1.00 |  |  |
| 2nd tertile | 1.11 | 0.86-1.43 |  | 1.05 | 0.56-1.98 |  |
| 3rd tertile | 1.18 | 0.89-1.56 |  | 1.25 | 0.64-2.45 |  |
| Sports/exercise |  |  |  |  |  | 0.978 |
| 1st tertile | 1.00 |  |  | 1.00 |  |  |
| 2nd tertile | 1.00 | 0.85-1.18 |  | 0.84 | 0.55-1.30 |  |
| 3rd tertile | 0.99 | 0.83-1.11 |  | 0.90 | 0.58-1.42 |  |
| Transportation |  |  |  |  |  | 0.148 |
| 1st tertile | 1.00 |  |  | 1.00 |  |  |
| 2nd tertile | 1.03 | 0.86-1.22 |  | 1.07 | 0.67-1.68 |  |
| 3rd tertile | 1.21 | 0.98-1.49 |  | 1.18 | 0.71-1.97 |  |

Abbreviation: PROM, premature rupture of membranes; OR, odds ratio; CI, confidential interval.

Odds ratios (95% CI) were calculated after adjustment of maternal age (continuous), occupations (farmers, workers, service worker, office and technical staff, housewife or unemployed), education level (senior high school or below, above senior high school),smoking status (never, ever, current) ,yearly income (＜50000，≥50000), pre‐pregnancy BMI (continuous), GDM during pregnancy, HDP during pregnancy, infant sex (male, female) and gestational weeks (continuous).
